# Whole-Genome Sequencing Re-Identifies Clinical “*Peptoniphilus harei*” Isolates as “*Peptoniphilus genitalis*”

**DOI:** 10.3390/pathogens15101073

**Published:** 2026-10-09

**Authors:** Liyan Mao, Chao Xu, Yuanxiang Zhao, Wanbo Fan, Chengxiu Hu, Siyu Zou, Huijuan Song, Hui Zhang, Hongbo Zhang, Ming Huang, Chi Zhang, Ziyong Sun, Zhongju Chen

**Affiliations:** 1 Department of Laboratory Medicine, Tongji Hospital, Tongji Medical College, Huazhong University of Science and Technology, Wuhan 430030, China; 2 Tongji Medical College, Huazhong University of Science and Technology, Wuhan 430030, China

**Keywords:** *Peptoniphilus genitalis*, misidentification, whole-genome sequencing, average nucleotide identity, MALDI-TOF MS, 16S rRNA gene, database limitation

## Abstract

Accurate species-level identification within the genus *Peptoniphilus* is challenging, yet routine methods, including biochemical identification, MALDI-TOF MS, and 16S rRNA gene sequencing, have not been systematically evaluated. We re-identified seven clinical isolates recovered between 2019 and 2025 from blood, ovarian abscess, diabetic foot, and skin and soft tissue, using VITEK-2 ANC, two MALDI-TOF MS platforms (Autof ms1000 and Bruker Biotyper), 16S rRNA gene sequencing, and whole-genome sequencing (WGS) combined with average nucleotide identity (ANI) analysis. VITEK-2 reported all seven isolates as *P. asaccharolyticus*; both MALDI-TOF platforms reported all as *P. harei* with high confidence (Autof ms1000 scores 9.299–9.638; Bruker Biotyper scores 2.008–2.227); and 16S rRNA sequencing could not reliably distinguish between *P. vaginalis* (98.60–99.23%) and *P. harei* (98.03–98.31%), as both identity ranges were close to the 98.65% species-level threshold and neither consistently exceeded it. WGS/ANI identified all seven isolates as *P. genitalis* (ANI 96.44–97.05% against the reference genome). All clinical isolates remained susceptible to β-lactams and vancomycin. Clindamycin resistance was common (6/7), and erythromycin showed a concordant pattern; the fluoroquinolones showed small inhibition zones in 5/7 isolates. However, erythromycin and the fluoroquinolones lacked a method-appropriate breakpoint and could not be formally categorized. The clinical distribution of *P. genitalis* is not limited by its name; conventional methods underrepresent this recently described species, and WGS/ANI should serve as the confirmatory method when identification conflicts with phenotypic observations.

## 1. Introduction

Bacteria of the genus *Peptoniphilus* are Gram-positive anaerobic cocci (GPAC) that commonly colonize the skin and the mucosal surfaces of the oral cavity, gastrointestinal tract, and urogenital tract [1,2]. In immunocompromised hosts or when local tissue barriers are breached, they can cause bacteremia, intra-abdominal infection, soft-tissue infection, and gynecological infection [3,4,5]. Although they make up a small share of clinical isolates overall, their pathogenic role in mixed anaerobic infections and in some monomicrobial infections is increasingly recognized [2,3,6].

The taxonomy of *Peptoniphilus* has remained unstable. The genus was originally segregated from *Peptostreptococcus* and now comprises more than 20 validly published species [7]. Several species are phenotypically very similar, and their 16S rRNA gene sequences differ too little for reliable discrimination, a persistent obstacle for routine identification [8,9,10]. The name “*Peptoniphilus vaginalis*” was proposed by Diop et al. but does not meet the requirements for valid publication under the International Code of Nomenclature of Prokaryotes and has never appeared on the List of Prokaryotic names with Standing in Nomenclature (LPSN) [6]. Only in 2024 did Abou Chacra et al. validly publish *Peptoniphilus genitalis* sp. nov. from a strain recovered from the human vaginal microbiome [11]. Around the same time, Wang et al. provisionally assigned a blood-culture isolate (SAHP1) from a cervical cancer patient to “*P. septimus*,” placing it phylogenetically close to *P. vaginalis* [12]. This nomenclatural and taxonomic instability has compounded the uncertainty of clinical identification.

Routine identification in clinical laboratories rests largely on commercial automated systems. Biochemical systems such as VITEK-2 compare the reactions of an anaerobic identification card (ANC) against an on-board database, whereas MALDI-TOF MS matches the protein fingerprint of an isolate against a reference spectral library. MALDI-TOF MS is accurate for most common pathogens, but its performance depends heavily on the breadth of the reference library and the quality of the spectra [13,14]. 16S rRNA gene sequencing is a widely used molecular approach; for some closely related species of *Peptoniphilus*, however, the inter-species sequence differences fall below the commonly used 98.65% species threshold, so the method cannot separate them reliably [8,15]. During routine diagnostics, we noticed that some clinical isolates reported as “*P. harei*” by MALDI-TOF MS were catalase-negative, which did not match the catalase-positive status described for *P. harei* in the literature and raised the possibility of misidentification.

Misidentification followed by correction has been documented across bacterial taxa. Members of the *Enterobacter cloacae* complex and several *Pseudomonas* species, for example, were misidentified for years while reference databases lagged behind and were recognized only after whole-genome sequencing (WGS) or average nucleotide identity (ANI) analysis [16,17]. The common thread is that when a database lacks the correct species, an identification system matches the unknown strain to the closest entry available and does so with high confidence, generating a systematic bias. The risk is presumably greater still for a genus like *Peptoniphilus*, whose taxonomy is still being settled.

The aim of this study was to establish the true species identity of seven clinical isolates reported by MALDI-TOF MS as “*P. harei*.” Specifically, we set out to (i) compare the identification results obtained by VITEK-2 ANC, MALDI-TOF MS, 16S rRNA gene sequencing, and WGS/ANI; (ii) determine the species identity of the isolates by WGS/ANI; (iii) identify why the routine methods misidentified them; and (iv) evaluate the value and the limits of phenotypic tests such as catalase and indole for distinguishing species within this genus.

## 2. Methods

### 2.1. Strain Collection and Ethics Approval

Seven anaerobic Gram-positive cocci isolated from clinical specimens at Tongji Hospital between January 2019 and December 2025 were collected retrospectively. Inclusion required a non-duplicate isolate identified by MALDI-TOF MS (Autof ms1000) as “*Peptoniphilus harei*” with a score of ≥9.0. The isolates originated from blood (*n* = 1), pus from an ovarian abscess (*n* = 1), diabetic foot infections (*n* = 4), and a skin pressure-ulcer infection (*n* = 1). The type strains *P. vaginalis* DSM 101742 (from the Leibniz Institute DSMZ-German Collection of Microorganisms and Cell Cultures, Braunschweig, Germany) and *P. harei* KCTC 5952 (from the Korean Collection for Type Cultures, KRIBB, Jeongeup, Republic of Korea) were used as controls. The study was approved by the ethics committee of Tongji Hospital (TJ-IRB20230836). The isolates were stored in 20% glycerol at −80 °C until use. As the isolates were obtained during routine clinical care and patient data were anonymized, the requirement for informed consent was waived by the ethics committee.

### 2.2. Conventional Biochemical and Phenotypic Identification

All seven clinical isolates and the two control strains were identified biochemically with the VITEK-2 system (bioMérieux, Marcy-l'Étoile, France) using the ANC card, following the manufacturer’s instructions. The ANC cards were incubated at 35.5 °C and read automatically by the VITEK-2 system after 8–12 h. Identification was interpreted according to the manufacturer’s confidence-level criteria. Catalase and indole tests were performed in duplicate for each isolate, with *Staphylococcus aureus* ATCC 25923 (catalase-positive) and *Escherichia coli* ATCC 25922 (indole-positive) as controls. Catalase was tested with 3% hydrogen peroxide: a fresh colony was smeared on a glass slide, a drop of 3% H_2_O_2_ was applied, and bubble formation was recorded. Indole was tested by the Kovacs method after growth in tryptone water at 35 °C for 48 h, judging a red ring at the reagent interface as positive. All clinical isolates and reference strains were tested in two independent culture batches.

### 2.3. MALDI-TOF MS

Isolates were identified in parallel on two MALDI-TOF MS platforms. On the Autof ms1000 (Autobio, Zhengzhou, China), fresh growth was prepared by the recommended formic acid/acetonitrile extraction, spotted onto the target plate, and matched against the on-board library (database v1.1.23) using Autof Analyzer v1.0.50. Results are expressed as a confidence score (0–10); scores ≥ 9.0 are considered reliable species-level identifications, 6.0–8.999 genus-level results, and <6.0 unreliable [18]. On the Bruker Biotyper (Bruker Daltonics, Bremen, Germany), the same formic acid/acetonitrile extraction was used, and spectra were matched against the MBT IVD library (version 2023). Results are expressed as a log score (0–3); scores ≥ 2.000 are considered reliable species-level identifications, 1.700–1.999 genus-level results, and <1.700 unreliable [19]. Quality control was performed weekly using *E. coli* ATCC 8739 and *S. aureus* ATCC 25923 as positive controls. Each isolate and reference strain was tested in two independent culture batches, and MALDI-TOF MS scores are reported as the range across the two batches.

### 2.4. 16S rRNA Gene Sequencing

Genomic DNA was extracted from fresh culture. The 16S rRNA gene was amplified by PCR with the universal primers 27F (5′-AGAGTTTGATCCTGGCTCAG-3′) and 1492R (5′-GGTTACCTTGTTACGACTT-3′). Each 50-μL PCR reaction contained 25 μL of 2× PCR master mix, 1 μL of each primer (10 μM), 2 μL of template DNA (approximately 50 ng), and nuclease-free water. Thermal cycling conditions were initial denaturation at 95 °C for 5 min; 30 cycles of 95 °C for 30 s, 55 °C for 30 s, and 72 °C for 90 s; and a final extension at 72 °C for 10 min. Negative controls (no template) were included in each PCR run. Amplicons were purified and Sanger-sequenced by Sangon Biotech (Shanghai, China). The resulting sequences were searched with the NCBI online BLASTn service (https://blast.ncbi.nlm.nih.gov/; default parameters) against the NCBI 16S ribosomal RNA sequences (Bacteria and Archaea) database (16S_ribosomal_RNA; accessed 19 August 2026). Percent identity, query coverage, and the number of gaps relative to the reference sequences were recorded. Percent identity values were compared with the 98.65% species-level threshold on an isolate-by-isolate basis. The 16S rRNA phylogenetic tree was constructed using the Neighbor-Joining method in MEGA 11 with 1000 bootstrap replicates. Only reference sequences longer than 1400 bp were included.

### 2.5. Whole-Genome Sequencing and Assembly

Genomic DNA was extracted from fresh culture using a Rapid Bacterial Genomic DNA Isolation Kit (Sangon Biotech, cat. no. B518225-0100), with lysozyme pretreatment for Gram-positive cell wall lysis. Library preparation was performed with the VAHTS Universal Pro DNA Library Prep Kit for Illumina (Sangon Biotech, cat. no. N608446). Paired-end sequencing (2 × 150 bp) was performed on the Illumina NovaSeq platform. Raw reads were quality-controlled using FastQC v0.11.2 and Trimmomatic v0.36, and reads with Q30 ≥ 85% were retained. Reads were assembled de novo with SPAdes v3.5.0, and gaps were filled using GapFiller v1.11 and PrInSeS-G v1.0.0. The assemblies were annotated with Prokka v1.10 and the NCBI Prokaryotic Genome Annotation Pipeline (PGAP v1.2.1), following the sequencing provider’s pipeline (report dated 16 April 2026; contract no. BADR2604958WH). Assembly statistics, such as total reads, Q30, GC content, total assembly length, contig number, N50, and the numbers of CDS, tRNA, and rRNA genes, were recorded for downstream analysis. Whole-genome sequencing was performed on all seven isolates. The raw reads have been deposited in the Sequence Read Archive under BioProject PRJNA1519044 (SRA: SRR40371697–SRR40371703).

### 2.6. Species Delineation and Phylogenetic Analysis

An all-versus-all ANI matrix was computed for the seven clinical genomes and six reference genomes: *P. genitalis* (GCF_023614525.1), *P. vaginalis* (GCF_900176605.1), *P. harei* (GCF_016726485.1), *P. tyrrelliae* (GCA_910592365.1), *P. faecalis* (GCA_012976305.1), and *P. asaccharolyticus* (GCA_016791605.1). Pairwise ANI values were computed using FastANI v1.33 with default settings (k-mer size 16, fragment length 3000 bp, minimum fraction 0.2) [20]. A 95% ANI threshold was used for species delineation. A UPGMA tree was built from the 1 − ANI distance matrix and visualized with iTOL v6.

### 2.7. Antimicrobial Susceptibility Testing

Seventeen antimicrobial agents were tested: penicillin, amoxicillin–clavulanate, piperacillin–tazobactam, ceftriaxone, cefepime, meropenem, imipenem, vancomycin, clindamycin, moxifloxacin, tetracycline, chloramphenicol, linezolid, erythromycin, levofloxacin, rifampin, and amikacin. MICs (mg/L) were determined by E-test (bioMérieux, France) for penicillin, ceftriaxone, meropenem, imipenem, vancomycin, and linezolid, and complemented by disk diffusion zones (mm) for the remaining agents. Testing was performed on Mueller–Hinton agar supplemented with 5% defibrinated sheep blood and incubated anaerobically at 35–37 °C for 48 h. Interpretive categories were assigned using the EUCAST v16.1 breakpoints for *Peptoniphilus* spp. as the primary reference [21]. EUCAST breakpoints were applied for penicillin, amoxicillin–clavulanate, piperacillin–tazobactam, meropenem, imipenem, vancomycin, and clindamycin. Where EUCAST provides no applicable breakpoint, CLSI breakpoints were used (CLSI M11, 9th ed., 2018, reaffirmed 2025, for anaerobic susceptibility testing methods [22]; CLSI M100-Ed35, 2025, for interpretive breakpoints [23]), but only when the breakpoint format matched the method performed: ceftriaxone (tested by MIC) was interpreted using the CLSI M100 anaerobic MIC breakpoint, applied by analogy to *Peptoniphilus*. For moxifloxacin, tetracycline, and chloramphenicol, which were tested by disk diffusion, CLSI provides no disk-diffusion breakpoints for anaerobes; because no method-appropriate breakpoint was available, only the measured zone diameters are reported, and no susceptible/intermediate/resistant category is assigned (indicated by “—”). Epidemiological cut-off values (ECOFFs) were also considered; however, EUCAST currently defines no ECOFF for *Peptoniphilus* spp. For cefepime, linezolid, erythromycin, levofloxacin, rifampin, and amikacin, neither EUCAST nor CLSI provides an anaerobic breakpoint applicable to *Peptoniphilus*; for these agents, only the measured values are reported, and no susceptible/intermediate/resistant category is assigned (indicated by “—”). Quality control was performed with *Bacteroides fragilis* ATCC 25285 and *Bacteroides thetaiotaomicron* ATCC 29741 according to CLSI recommendations. *P. vaginalis* DSM 101742 and *P. harei* KCTC 5952 were tested in parallel for method validation.

## 3. Results

### 3.1. Patient and Isolates

Seven patients were enrolled between January 2019 and December 2025; their clinical characteristics are summarized in Table 1. The patients came from the departments of dermatology, endocrinology, reproductive medicine, and cardiology; most (4/7, 57.1%) were from endocrinology. Ages ranged from 31 to 87 years (median 53), and five patients (71.4%) were male. At admission, two patients (28.6%) were febrile, one (14.3%) reported cough, fatigue, and loss of appetite, four (57.1%) had lost weight, and five (71.4%) had pain at the site of infection. Every patient had at least one underlying condition, most commonly diabetes (5/7, 71.4%) and hypertension (1/7, 14.3%). The clinical diagnoses comprised diabetic foot infection (*n* = 4), bloodstream infection (*n* = 1), ovarian abscess (*n* = 1), and skin and soft-tissue infection (*n* = 1). Laboratory findings included a markedly elevated white blood cell count (>20 × 10^9^/L) in two patients and a C-reactive protein level above 50 mg/L in three patients (peak 285.1 mg/L). Co-pathogens were recovered from the same site in three patients (42.9%): staphylococci, Enterobacterales, fungi, and other anaerobes; in two further patients, only organisms classified as contaminants (*Staphylococcus epidermidis*) were recovered from the site. All patients received antimicrobial therapy (mainly fluoroquinolones, vancomycin, and metronidazole) and recovered; the median time to recovery was 7 days (range 4–15 days).

### 3.2. Routine Identification and Phenotypic Testing

After subculture, the seven clinical isolates appeared by Gram staining as Gram-positive cocci in pairs, single cells, or clusters (Figure S1). All seven isolates and the two control strains (*P. vaginalis* DSM 101742 and *P. harei* KCTC 5952) were subjected in parallel to biochemical identification, MALDI-TOF MS, catalase, and indole testing; 16S rRNA gene sequencing was performed on the seven clinical isolates (the reference strains are cited by their type-strain 16S accessions; Table 2 note b). The results are summarized in Table 2.

VITEK-2 with the ANC card identified all nine strains (seven clinical isolates and two controls) as *P. asaccharolyticus* with 98–99% confidence (Table S1). The two MALDI-TOF platforms agreed closely: Autof ms1000 reported all nine strains as *P. harei* at the species level (clinical isolates, scores 9.008–9.638; reference strains, 8.022–9.516; threshold ≥ 9.0). Bruker Biotyper reported the seven clinical isolates as *P. harei*/*P. indolicus*, with scores ranging from 1.945 to 2.227; several batches fell below the species-level threshold of 2.000. For the reference strains, Bruker scores ranged from 1.440 to 1.476 for DSM 101742 (all below the genus-level threshold of 1.700) and from 1.699 to 1.906 for KCTC 5952 (straddling the genus-level threshold). Neither reference strain reached the species level. All seven clinical isolates and DSM 101742 were catalase-negative, whereas KCTC 5952 was catalase-positive. The Kovacs indole test was positive for all seven clinical isolates and two type strains (Table 2).

**Figure 1:**
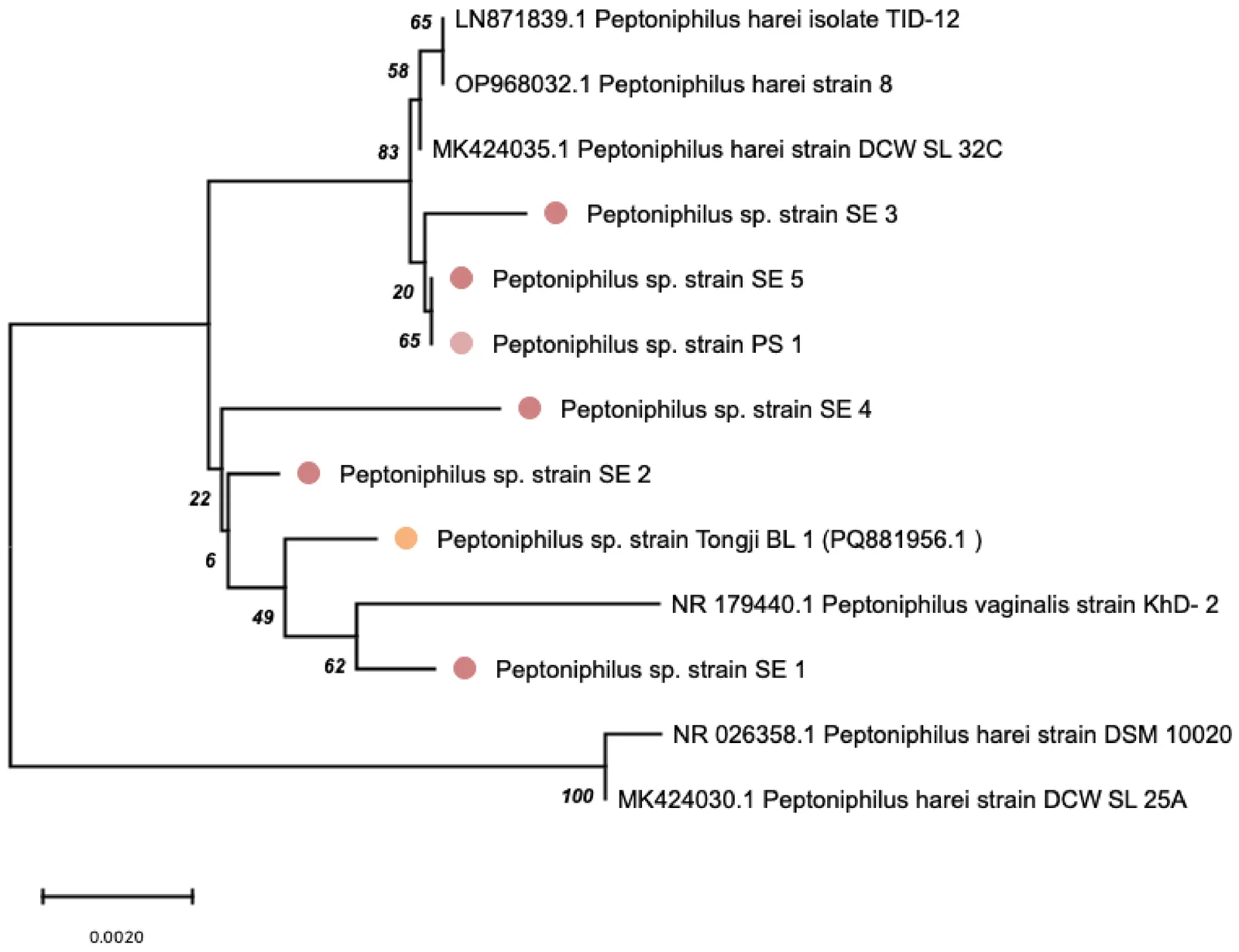
Phylogenetic tree of the genus *Peptoniphilus* based on 16S rRNA gene sequences. The tree was constructed using the Neighbor-Joining method in MEGA 11 software. Bootstrap values (based on 1000 replicates) are shown at the branch nodes. The scale bar indicates the genetic distance per nucleotide site. Only reference sequences exceeding 1400 bp in length were included. The seven clinical isolates are marked with colored dots (●) according to specimen type: dark red for secretion, light red for pus, and orange for blood, and labeled with their strain identifiers (SE 1–SE 5, BL 1, and PS 1). Reference sequences are shown with their GenBank accession numbers. The clinical isolates showed 98.60–99.23% similarity to *P. vaginalis* (NR_179440.1) and 98.03–98.31% similarity to *P. harei* (NR_026358.1) and could not be reliably distinguished from these closely related species by 16S rRNA sequencing.

16S rRNA gene sequencing (sequences deposited in GenBank under PX884293–PX884298 and PQ881956) returned 98.60–99.23% identity with *P. vaginalis* (NR_179440.1) and 98.03–98.31% with *P. harei* (NR_026358.1) for all seven isolates (Table S2). When compared with the 98.65% species-level threshold on an isolate-by-isolate basis, some values for *P. vaginalis* fell below the threshold while others exceeded it, whereas all values for *P. harei* fell below the threshold; neither range consistently exceeded the threshold. No *P. genitalis* reference sequence was present in the NCBI 16S database at the time of the search (19 August 2026), so this species could not be included in the comparison. To clarify the phylogenetic position, we built a 16S rRNA gene tree (Figure 1). Several of our isolates (*Peptoniphilus* sp. SE1, SE2, SE4, and Tongji BL 1) clustered tightly with the *P. vaginalis* reference NR_179440.1 in a distinct branch, whereas SE3, SE5, and PS1 grouped with certain reported *P. harei* strains (e.g., DCW SL 32C) in a separate branch. This inconsistent clustering, together with the overlapping identity ranges, indicates that 16S rRNA sequencing alone could not reliably assign these isolates to either species.

### 3.3. WGS/ANI Confirmation of P. genitalis

**Figure 2:**
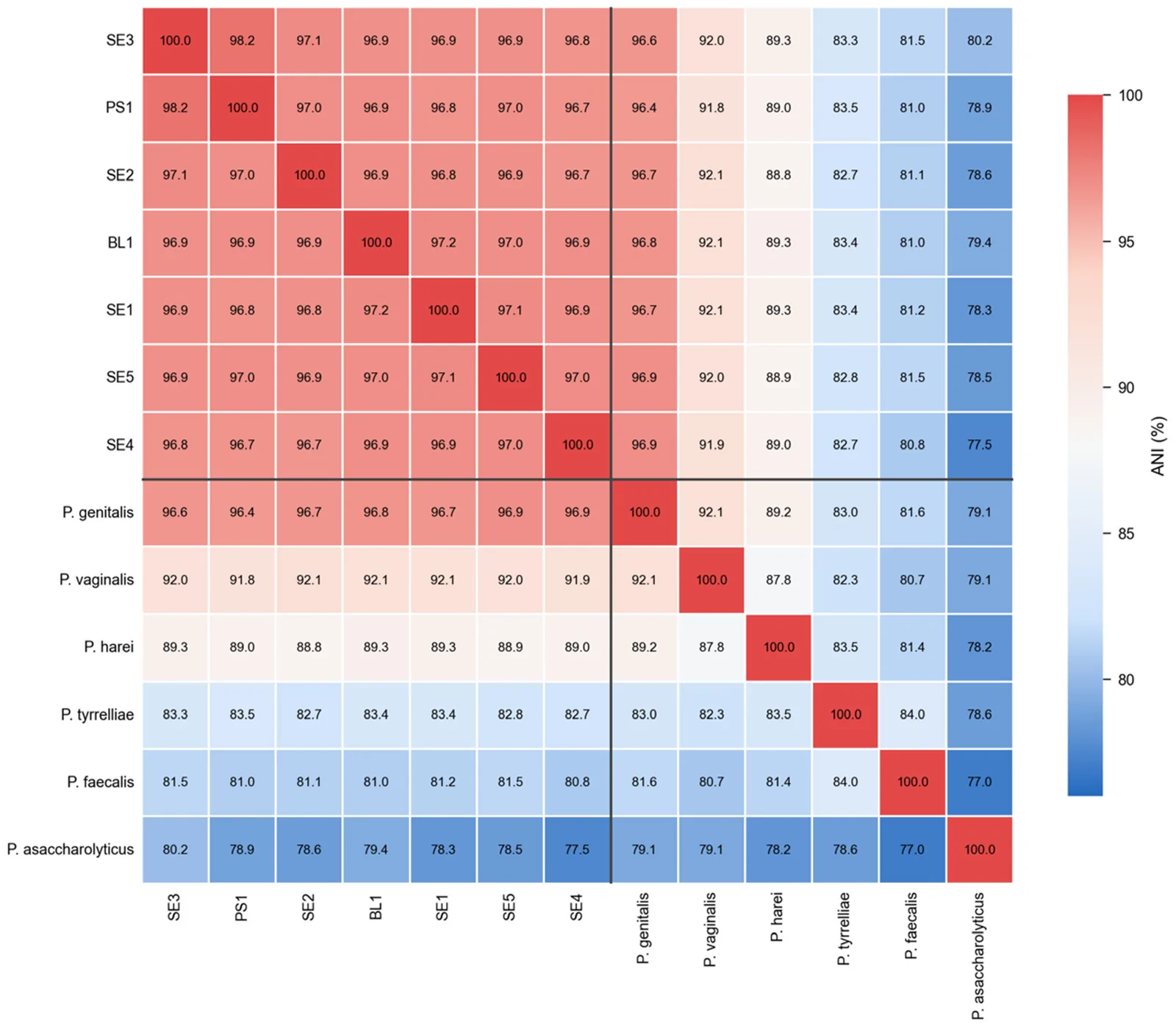
Heatmap of average nucleotide identity (ANI) among 13 genomes of the genus *Peptoniphilus***.** Colors ranging from red to blue represent high to low ANI values (%). The seven clinical isolates (SE 1–SE 5, BL 1, and PS 1) showed ANI values of 96.44–97.05% against the *P. genitalis* reference genome (GCA_023614525.1), exceeding the 95% species delineation threshold. ANI values against *P. vaginalis* (91.84–92.29%), *P. harei* (88.86–89.32%), and the other reference species (77.5–83.5%) were clearly lower. Values were calculated using FastANI and visualized using Matplotlib v3.10.9.

**Figure 3:**
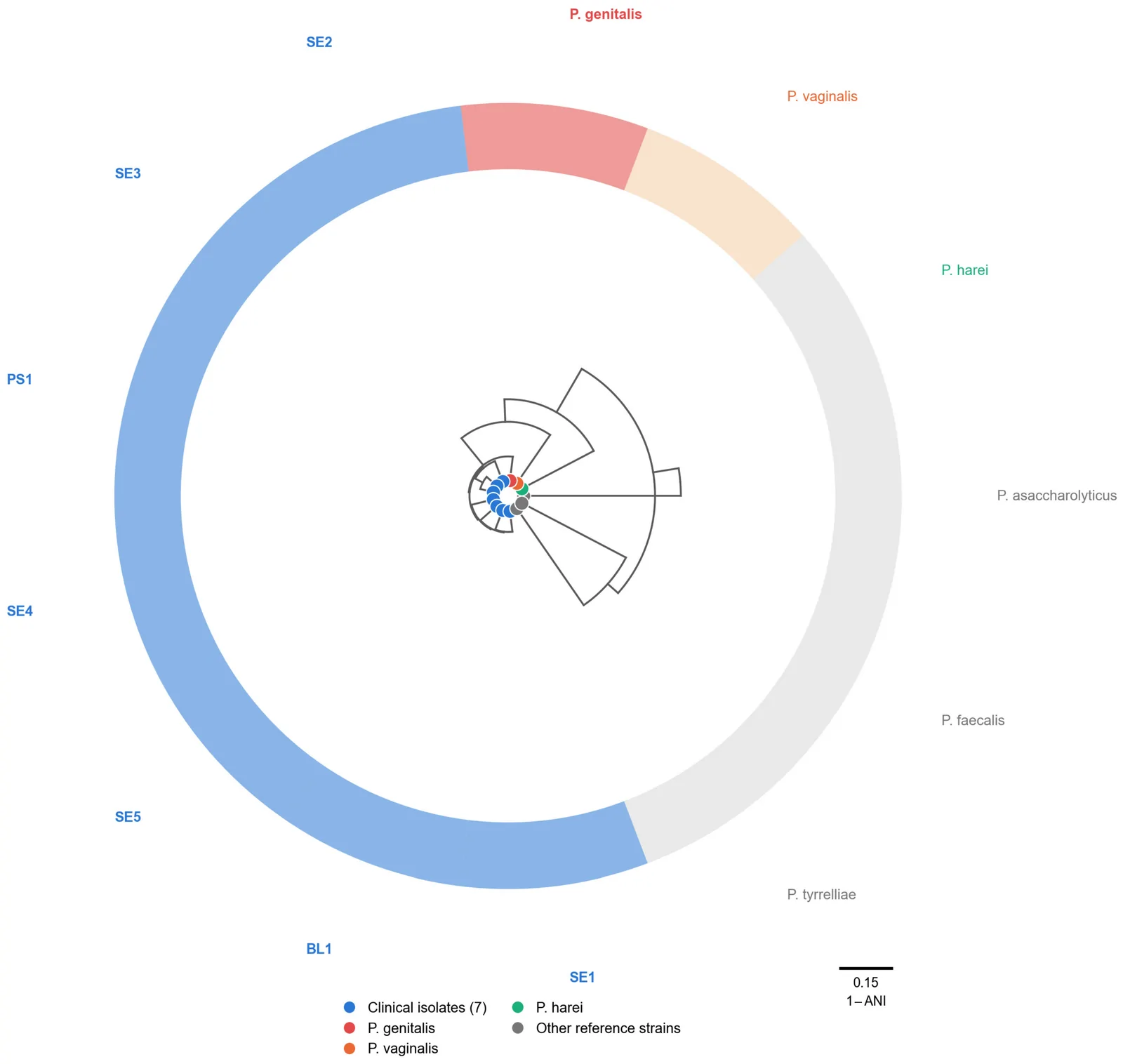
UPGMA circular phylogenetic tree of the genus *Peptoniphilus* based on the 1−ANI distance matrix. The seven clinical isolates (blue), the *P. genitalis* reference genome (red), *P. vaginalis* (orange), and other reference species (gray) are color-coded. The seven clinical isolates cluster with *P. genitalis* in the same clade (red branch), forming a cluster adjacent to *P. vaginalis* but clearly separated from *P. harei*. The outer color ring indicates the taxonomic assignment of each strain. The tree was visualized using iTOL v6.

Sequencing and assembly produced good-quality data for all seven isolates (N50, 79–336 kb; assembly statistics in Table S3). The raw reads have been deposited in the Sequence Read Archive (SRA) under accession numbers SRR40371697–SRR40371703, and the assembled genomes are available under BioProject PRJNA1519044 (with individual GenBank accession numbers to be assigned upon genome submission). ANI analysis (Figure 2; Table S4) showed pairwise ANI values of 96.71–98.20% among the seven isolates and 96.44–97.05% against the *P. genitalis* reference genome (GCF_023614525.1), exceeding the 95% species threshold, while values against *P. vaginalis* (91.84–92.29%), *P. harei* (88.86–89.32%), and the other reference species (77.5–83.5%) were clearly lower. In the UPGMA tree constructed from 1 − ANI distances (Figure 3), the seven isolates formed a single clade with *P. genitalis*, adjacent to *P. vaginalis* and well separated from *P. harei*. Based on the combined ANI and phylogenetic evidence, all seven clinical isolates were identified as *P. genitalis*.

### 3.4. Antimicrobial Susceptibility

MICs and zone diameters for the 17 agents are given in Table 3. All seven clinical isolates were susceptible to penicillin (MIC 0.006–0.25 mg/L), the β-lactam/β-lactamase-inhibitor combinations (amoxicillin–clavulanate, piperacillin–tazobactam), ceftriaxone, the carbapenems (imipenem, meropenem), and vancomycin.

For clindamycin, the EUCAST zone-diameter breakpoint was applicable, and 6/7 isolates (85.7%) were resistant. For the remaining agents, no method-appropriate breakpoint was available for disk diffusion, or the applicable breakpoint format did not match the method performed, so zone diameters are reported descriptively without interpretive categories. These included cefepime (11–38 mm), erythromycin (6–21 mm), levofloxacin (6–21 mm), moxifloxacin (6–32 mm), tetracycline (10–19 mm), rifampin (30–40 mm), amikacin (10–21 mm), and chloramphenicol (25–32 mm). Linezolid was tested by MIC and also lacked an applicable breakpoint (0.125–0.5 mg/L).

Several descriptive patterns are noteworthy. Levofloxacin and moxifloxacin showed small zones (6 mm) in 5/7 isolates, with only the bloodstream isolate BL 1 and the reference strain DSM 101742 showing larger zones (21–32 mm). Erythromycin and clindamycin results were concordant: 6/7 isolates showed 6 mm zones for both agents, except for the bloodstream isolate BL 1, which showed a 21 mm zone for erythromycin and a 26 mm zone for clindamycin (susceptible). Rifampin and chloramphenicol produced consistently large zones (30–40 mm and 25–32 mm, respectively) across all isolates. Linezolid MICs were uniformly low (0.125–0.5 mg/L). Because no method-appropriate breakpoint was available, these values could not be formally categorized.

*P. vaginalis* DSM 101742 and *P. harei* KCTC 5952 were tested in parallel; both were susceptible to most β-lactams, and both were resistant to tetracycline. Table S5 compares the susceptibility profile of the clinical isolates with that of the *P. genitalis* type strain (Marseille-Q7072T; [11]) and with previously published data.

## 4. Discussion

Using WGS and ANI, we re-identified seven clinical isolates reported by MALDI-TOF MS as *P. harei*; all proved to be *P. genitalis*. The finding echoes the single observation of Wang et al., whose blood-culture isolate SAHP1 was likewise misreported by MALDI-TOF MS before genome-based taxonomy placed it in the *P. genitalis*/*P. septimus* group, and it suggests that database-dependent methods may misidentify *Peptoniphilus* more often than is recognized [11,12]. Our series extends that case to several infection sites and shows that current commercial databases and 16S rRNA gene sequencing both fail to recognize the species.

VITEK-2 identified all seven clinical isolates and both control strains as *P. asaccharolyticus* (98–99% confidence), in direct conflict with the WGS result and pointing to a clear gap in the coverage of *Peptoniphilus* in commercial databases. Catalase was negative in all seven clinical isolates and in *P. vaginalis* DSM 101742 but positive in *P. harei* KCTC 5952, consistent with the description by Diop et al. [6,24]. This test can help separate *P. vaginalis* from *P. harei*. However, because catalase is also negative in *P. genitalis*, a negative result provides no species-level evidence for identifying an isolate as *P. genitalis*; its diagnostic value in this workflow is therefore limited to distinguishing *P. vaginalis* from *P. harei*, and it cannot be used to identify *P. genitalis*. Indole was positive in all nine isolates (seven clinical isolates and two reference strains), indicating that this test does not discriminate among these closely related species. Species identification within *Peptoniphilus* therefore cannot rest on biochemical reactions or a single phenotypic test; clinical laboratories should at least report these organisms to the genus level and turn to molecular or genomic methods for clinically relevant cases.

The two MALDI-TOF platforms agreed closely, and this agreement is precisely the problem: all seven clinical isolates met the criteria for reliable species-level identification on both platforms (Autof ms1000 scores 9.299–9.638; Bruker Biotyper 2.008–2.227), yet every report named *P. harei*. The *P. vaginalis* type strain DSM 101742 was likewise reported as *P. harei* (species level on Autof ms1000, 9.516; genus level on Bruker Biotyper, 1.476), indicating that the problem lies not with the strains or the procedure but with the absence of accurate reference spectra for *P. vaginalis* in commercial databases. Routine MALDI-TOF MS libraries generally lag behind taxonomy, and *P. genitalis* was validly published only in 2024 [11]; when the correct species is absent, the system matches the unknown spectrum to the closest entry. Here, *P. harei* was identified with high confidence. Laboratories should therefore treat a high score with reserve: it does not validate an identification, and results that represent a rare species match or contradict the phenotype deserve scrutiny, with WGS as the arbiter. We suggest that manufacturers add reference spectra for newly described species to narrow the gap between taxonomy and practice.

16S rRNA gene sequencing also failed to identify the isolates. No *P. genitalis* reference sequence was present in the NCBI 16S database at the time of analysis, so BLAST (NCBI online BLASTn service; accessed 19 August 2026) could only choose between *P. vaginalis* (98.60–99.23% identity) and *P. harei* (98.03–98.31%). This inconsistent clustering, together with the overlapping identity ranges, indicates that 16S rRNA sequencing alone could not reliably assign these isolates to either species. Some isolates clustered with *P. harei* in the 16S tree, although WGS/ANI placed them in *P. genitalis*. This discordance confirms how little phylogenetic signal these sequences carry for species-level assignment. Above 98% similarity, tree topologies are often driven by noise or a few informative positions rather than by genuine evolutionary relationships [25,26,27]. This limitation is well documented in taxonomically complex groups such as *Pseudomonas* and the *Enterobacter cloacae* complex [16,17]. Quality issues in the public databases add further uncertainty, and the taxonomy of *Peptoniphilus* is itself still shifting: genome-based analyses divide the genus into eight lineages, with some species proposed for transfer to new genera. Species identification from a single 16S gene therefore has an inherent structural weakness, and we recommend WGS/ANI as the final arbiter when 16S results conflict with the phenotype or mass spectrometry.

Whole-genome sequencing and ANI provided the decisive evidence. All seven isolates shared 96.44–97.05% ANI with *P. genitalis* (GCF_023614525.1)—above the 95% threshold—and clearly lower ANI with *P. vaginalis* (91.84–92.29%) and *P. harei* (88.86–89.32%), confirming three distinct species; the UPGMA tree placed the isolates with *P. genitalis*, separate from *P. harei*. The misreporting of the *P. vaginalis* type strain DSM 101742 by MALDI-TOF MS again points to the lack of accurate reference spectra for *P. vaginalis* in commercial databases. Taxonomically, “*P. vaginalis*” was never validly published, whereas *P. genitalis* was, so priority rests with *P. genitalis*. Abou Chacra et al. further noted that their type strain Marseille-Q7072T and the “*P. septimus*” strain SAHP1 of Wang et al. belong to the same species; “*P. septimus*” has neither been deposited in a culture collection nor validly published, making *P. genitalis* the correct name [11,12].

In routine clinical microbiology, species-level identification of *Peptoniphilus* is usually not required. There are technical and clinical reasons: the species are hard to tell apart even with combined methods, and *Peptoniphilus* usually participates in mixed infections as an accompanying organism, so the species name matters little for treatment decisions. What our findings add is a different kind of risk. When the reference databases that routine methods rely on lack a species or carry a mislabeled entry for it, the result can point to the wrong species with high confidence—concealing the true distribution of a newly described species and, for an individual patient, potentially creating expectations about the susceptibility profile that are not supported by the actual species identity. WGS/ANI is therefore not a front-line tool to replace routine methods but an arbiter for them: a way to settle species identity when identification conflicts with the phenotype or involves a rare organism, and a basis for updating the databases.

Our data also add to the clinical picture of *P. genitalis*. Although the species was first isolated from a vaginal specimen and named accordingly, the seven isolates came from blood, an ovarian abscess, diabetic foot infections, and skin and soft tissue, and the patients included both men and women. The name should not be taken to confine the organism’s pathogenic potential: *P. genitalis* is an opportunistic pathogen that can infect multiple sites. Laboratories should not let the name bias interpretation, and clinicians should judge the isolate in the light of the infection site and clinical context.

Accurate species-level identification within the genus *Peptoniphilus* has direct implications for empirical therapy, because susceptibility patterns can differ among species. Guérin et al., in a multicenter study of 133 Gram-positive anaerobic cocci, including 30 *Peptoniphilus* isolates, showed that clindamycin resistance is common and often mediated by *erm* genes, and that susceptibility to several agents varies across species [28]. In the present study, *P. genitalis* clinical isolates were frequently resistant to clindamycin, whereas the *P. harei* type strain KCTC 5952 was susceptible (zone 26 mm). This contrast illustrates that correct species identification is not merely a taxonomic exercise but can directly inform treatment decisions, because a misidentified isolate may lead to expectations about susceptibility that do not apply to the actual pathogen.

All seven clinical isolates were susceptible to penicillin, the β-lactam/β-lactamase-inhibitor combinations, ceftriaxone, the carbapenems, and vancomycin. The profile is consistent with previously reported data for *P. harei* and *P. vaginalis* and supports these agents as reasonable empirical options for infections caused by this genus [4,6,11,29,30,31,32,33]. Among the agents for which an applicable breakpoint was available, clindamycin resistance was observed in six of seven isolates. This finding contrasts with published data for *P. harei* isolates of Cobo et al. [4,29], the *P. genitalis* type strain of Abou Chacra et al. [11], and our own *P. harei* KCTC 5952 control, all of which were susceptible. Because the molecular basis of resistance was not investigated and clonality was not assessed, the possibility of acquired resistance remains speculative. Nevertheless, the distribution of this phenotype across isolates from different patients and infection sites is not easily explained by a species-level trait alone, although this interpretation requires confirmation. Pending such confirmation, these preliminary observations suggest that clindamycin should be used with caution in empirical therapy for infections caused by this genus.

The possibility that susceptibility patterns may differ by infection site is intriguing but is based on very few isolates. The bloodstream isolate appeared susceptible to clindamycin and showed larger inhibition zones for several agents than isolates from secretions or abscesses. With only one bloodstream isolate and a small overall sample, however, this observation could be due to chance, local selection pressure, or other unmeasured factors, and it should not be overinterpreted. A similar pattern was observed for erythromycin: six of seven isolates showed small zones (6 mm), whereas the bloodstream isolate showed a larger zone (21 mm), consistent with the concordance between erythromycin and clindamycin results described above. The fluoroquinolones followed a comparable trend in most isolates, with five of seven showing zones of 6 mm for both levofloxacin and moxifloxacin. Whether these patterns reflect a common resistance mechanism or site-related selection cannot be determined from our data, but they may warrant further investigation. If such site-related differences were real, accurate species identification would still matter, because misidentification could potentially create expectations about susceptibility based on the wrong species. Wan et al. reported a *P. harei* bloodstream isolate that was susceptible to all agents tested, including clindamycin [32]. Had our bloodstream isolate been reported as *P. harei*, clinicians might have expected a similarly susceptible profile, without considering the possibility of acquired resistance in the clinical isolate. This scenario, however, is hypothetical and is not supported by clinical outcome data. Two caveats apply. The data are entirely in vitro, and *Peptoniphilus* lacks complete species-specific breakpoints in both CLSI and EUCAST, so for several agents only measured values could be reported; the profiles should be treated as preliminary references rather than predictors of clinical outcome.

This study has several limitations. It is single-center and retrospective, so the conclusions are limited in generalizability; the seven isolates cover several infection types, and the WGS evidence is uniform, but the findings need confirmation in larger, multicenter prospective studies. We did not examine the molecular basis of the presumed acquired resistance (e.g., *erm* genes, efflux pumps), nor did we perform a clonality analysis of the seven isolates; the inference rests on the distribution of the phenotype and on comparison with the literature. Confirmation of independent acquisition will require molecular characterization and core-genome relatedness analysis in future studies. Finally, the pathogenicity and specific role of *P. genitalis* in mixed infections remain to be investigated. Within these limits, this case series of a newly described species documents a weakness of database-dependent identification that was uniform across the cohort and provides a basis for larger epidemiological and resistance-mechanism studies.

## 5. Conclusions

Whole-genome sequencing combined with ANI re-identified all seven clinical isolates reported by MALDI-TOF MS as *P. harei*, and every one of them proved to be *P. genitalis*. The routine methods—VITEK-2 ANC, MALDI-TOF MS on two platforms, and 16S rRNA gene sequencing—each failed to give a correct species identification; the misreporting stems from the incomplete coverage of this newly described species in commercial and public sequence databases and from the inherently limited resolving power of 16S rRNA gene sequences among close relatives. MALDI-TOF MS scores were high even though the results pointed to the wrong species throughout, a reminder that laboratories should treat identifications of unusual species with caution. Among the phenotypic tests, catalase helped separate *P. vaginalis* from *P. harei* but could not distinguish *P. genitalis* from *P. vaginalis*. All clinical isolates were susceptible to β-lactams and vancomycin. Clindamycin resistance was common (6/7), and erythromycin showed a concordant pattern; the fluoroquinolones showed small inhibition zones in 5/7 isolates, although these agents could not be formally categorized because no method-appropriate breakpoint was available. The clinical distribution of *P. genitalis* is not bound by its name: it can cause bloodstream, diabetic foot, ovarian abscess, and skin and soft-tissue infections. We suggest that database manufacturers add *P. genitalis* reference spectra promptly and that clinical laboratories turn to WGS/ANI for confirmation whenever a *Peptoniphilus* isolate is reported with results that conflict with the phenotype.

## Figures and Tables

**Table 1:** Clinical characteristics of seven patients with *Peptoniphilus genitalis* isolates.

Variable	BL 1	PS 1	SE 1	SE 2	SE 3	SE 4	SE 5
Demographics							
Age (years)	55	31	59	53	87	50	42
Sex	Male	Female	Male	Male	Female	Male	Male
Clinical features							
Fever	Yes	Yes	No	No	No	No	No
Cough	No	No	No	Yes	No	No	No
Fatigue	No	No	No	Yes	No	No	No
Anorexia	No	No	No	Yes	No	No	No
Weight loss	Yes	No	No	Yes	Yes	No	Yes
Pain ^a^	Yes (foot)	No	Yes (foot)	No	Yes (foot)	Yes (foot)	Yes (foot)
Underlying conditions							
Hypertension	No	No	Yes	No	No	No	No
Diabetes mellitus	Yes	No	Yes	No	Yes	Yes	Yes
Smoking	Yes	No	No	No	No	No	No
Other	No	Cephalosporin allergy	No	COPD; DCM	No	S/P gastric perforation surgery	No
Infection							
Source	Blood	Ovary	Foot	Skin	Foot	Foot	Foot
Diagnosis	Bacteremia	Ovarian abscess	Diabetic foot infection	Pressure sore infection	Diabetic foot infection	Diabetic foot infection	Diabetic foot infection
Laboratory findings							
WBC (×10^9^/L)	14.57	7.34	26.35	20.24	6.7	5.84	6.16
Neutrophils (×10^9^/L)	12.88	5.83	22.71	19.46	4.52	4.06	3.80
CRP (mg/L)	285.1	68.8	14.5	70.1	0.9	5.6	3.4
PCT (ng/mL) ^b^	0.87	<0.05	0.16	Not tested	<0.05	Not tested	Not tested
Co-isolates							
Same site ^c^	Sep (contaminant)	None	None	Pmi; Kpn; Sau; Tas	Sep (contaminant); Cpa	Sau	Bfr
Other site ^c^	Pmi (ulcer exudate)	None	None	None	None	None	None
Antimicrobial therapy ^d^	Quinolones; ornidazole; linezolid	Quinolones	Quinolones; cephalosporin; itraconazole	Carbapenems; vancomycin; daptomycin	Piperacillin–tazobactam	Miconazole (topical)	Piperacillin–tazobactam; cephalosporin
Outcome	Recovery	Recovery	Recovery	Recovery	Recovery	Recovery	Recovery
Time to recovery (days)	5	7	15	15	10	6	4

Abbreviations: BL, blood; PS, pus; SE, secretion; WBC, white blood cell; CRP, C-reactive protein; PCT, procalcitonin; COPD, chronic obstructive pulmonary disease; DCM, dilated cardiomyopathy; S/P, status post; Sep, *Staphylococcus epidermidis*; Pmi, *Proteus mirabilis*; Kpn, *Klebsiella pneumoniae*; Sau, *Staphylococcus aureus*; Tas, *Trichosporon asahii*; Cpa, *Candida parapsilosis*; Bfr, *Bacteroides fragilis*. ^a^ Pain denotes pain localized at the infection site; “foot” indicates pain attributable to the diabetic foot infection. ^b^ PCT was not tested in patients SE 2, SE 4, and SE 5. ^c^ “None” indicates no co-isolate at the indicated site. Co-isolates judged as contaminants by clinical and laboratory criteria are labelled “(contaminant)”. ^d^ Antimicrobial therapy refers to agents administered for the index infection.

**Table 2:** Identification of seven clinical isolates and two reference strains by routine methods and whole-genome sequencing.

Isolate (Source)	16S rRNA Gene Sequencing	VITEK-2 ANC Card	MALDI-TOF MS (Autof ms1000) ^c^	MALDI-TOF MS (Bruker Biotyper) ^d^	Catalase	Indole	WGS-Based Identification (ANI)
BL 1 (blood)	*P. vaginalis* (database-limited) ^a^	*P. asaccharolyticus*	*P. harei* (9.167–9.299)	*P. harei/P. indolicus* (2.082–2.129)	Negative	Positive	*P. genitalis*
PS 1 (pus)	*P. vaginalis* (database-limited) ^a^	*P. asaccharolyticus*	*P. harei* (9.361–9.567)	*P. harei/P. indolicus* (1.968–2.008)	Negative	Positive	*P. genitalis*
SE 1 (secretion)	*P. vaginalis* (database-limited) ^a^	*P. asaccharolyticus*	*P. harei* (9.341–9.502)	*P. harei/P. indolicus* (2.205–2.208)	Negative	Positive	*P. genitalis*
SE 2 (secretion)	*P. vaginalis* (database-limited) ^a^	*P. asaccharolyticus*	*P. harei* (9.565–9.584)	*P. harei/P. indolicus* (1.945–2.145)	Negative	Positive	*P. genitalis*
SE 3 (secretion)	*P. vaginalis* (database-limited) ^a^	*P. asaccharolyticus*	*P. harei* (9.167–9.360)	*P. harei/P. indolicus* (2.078–2.187)	Negative	Positive	*P. genitalis*
SE 4 (secretion)	*P. vaginalis* (database-limited) ^a^	*P. asaccharolyticus*	*P. harei* (9.008–9.428)	*P. harei/P. indolicus* (2.122–2.227)	Negative	Positive	*P. genitalis*
SE 5 (secretion)	*P. vaginalis* (database-limited) ^a^	*P. asaccharolyticus*	*P. harei* (9.618–9.638)	*P. harei/P. indolicus* (2.129–2.223)	Negative	Positive	*P. genitalis*
DSM 101742 (=KHD2, *P. vaginalis* type strain)	*P. vaginalis* (LN907856) ^b^	*P. asaccharolyticus*	*P. harei* (8.022–9.516)	*P. harei/P. indolicus* (1.440–1.476)	Negative	Positive	*P. vaginalis* (GCF_900176605.1) ^e^
KCTC 5952 (=DSM 10020, *P. harei* type strain)	*P. harei* (Y07839) ^b^	*P. asaccharolyticus*	*P. harei* (9.051–9.073)	*P. harei/P. indolicus* (1.699–1.906)	Positive	Positive	*P. harei* (GCF_900454685.1) ^e^

Abbreviations: WGS, whole-genome sequencing; ANI, average nucleotide identity; ANC, anaerobic and Corynebacterium; MALDI-TOF MS, matrix-assisted laser desorption/ionization time-of-flight mass spectrometry. ^a^ The top 16S rRNA gene BLAST hit for the seven clinical isolates was *P. vaginalis* because no 16S rRNA reference sequence of *P. genitalis* was present in the NCBI 16S database at the time of testing (database coverage limitation); per-isolate identity and coverage values are provided in Table S1. ^b^ 16S rRNA gene sequence accession numbers of the type strains; 16S sequencing of the reference strains was not performed in this study. ^c^ MALDI-TOF MS (Autof ms1000, database v1.1.23): scores ≥ 9.0 for species-level, 6.0–8.999 for genus-level, <6.0 unreliable. ^d^ MALDI-TOF MS (Bruker Biotyper, database version 2023): scores ≥ 2.000 for species-level, 1.700–1.999 for genus-level, <1.700 unreliable. ^e^ The WGS-based identification of the reference strains corresponds to their type-strain reference genomes (GCF_900176605.1, *P. vaginalis* strain KhD-2; GCF_900454685.1, *P. harei* strain DSM 10020); whole-genome sequencing of the reference strains was not performed in this study.

**Table 3:** Antimicrobial susceptibility of the nine *Peptoniphilus* isolates (seven clinical isolates and two reference strains).

Antimicrobial Agent	Method	BL 1	PS 1	SE 1	SE 2	SE 3	SE 4	SE 5	DSM 101742	KCTC 5952
Penicillin (P)	MIC (mg/L)	0.25 (S)	0.19 (S)	0.032 (S)	0.25 (S)	0.094 (S)	0.19 (S)	0.006 (S)	0.24 (S)	0.094 (S)
Amoxicillin–clavulanate (AMC)	Zone (mm)	38 (S)	38 (S)	38 (S)	38 (S)	42 (S)	38 (S)	42 (S)	40 (S)	40 (S)
Piperacillin–tazobactam (TZP)	Zone (mm)	40 (S)	48 (S)	42 (S)	46 (S)	44 (S)	42 (S)	48 (S)	42 (S)	40 (S)
Ceftriaxone (CRO)	MIC (mg/L)	0.38 (S)	0.094 (S)	0.125 (S)	0.38 (S)	0.125 (S)	0.38 (S)	0.125 (S)	0.094 (S)	0.125 (S)
Cefepime (FEP)	Zone (mm)	24 (—)	32 (—)	28 (—)	11 (—)	34 (—)	28 (—)	28 (—)	30 (—)	38 (—)
Meropenem (MEM)	MIC (mg/L)	0.008 (S)	0.012 (S)	0.006 (S)	0.04 (S)	0.008 (S)	0.006 (S)	0.006 (S)	0.004 (S)	0.008 (S)
Imipenem (IPM)	MIC (mg/L)	0.012 (S)	0.024 (S)	0.008 (S)	0.008 (S)	0.012 (S)	0.008 (S)	0.008 (S)	0.006 (S)	0.032 (S)
Vancomycin (VAN)	MIC (mg/L)	0.125 (S)	0.125 (S)	0.19 (S)	0.125 (S)	0.125 (S)	0.125 (S)	0.125 (S)	0.125 (S)	0.094 (S)
Linezolid (LNZ)	MIC (mg/L)	0.25 (—)	0.25 (—)	0.5 (—)	0.125 (—)	0.25 (—)	0.25 (—)	0.5 (—)	0.25 (—)	0.25 (—)
Erythromycin (E)	Zone (mm)	21 (—)	6 (—)	6 (—)	6 (—)	6 (—)	6 (—)	6 (—)	16 (—)	20 (—)
Clindamycin (DA)	Zone (mm)	26 (S)	6 (R)	6 (R)	6 (R)	13 (R)	6 (R)	6 (R)	26 (S)	28 (S)
Levofloxacin (LEV)	Zone (mm)	21 (—)	14 (—)	6 (—)	6 (—)	6 (—)	6 (—)	6 (—)	16 (—)	15 (—)
Moxifloxacin (MXF)	Zone (mm)	32 (—)	30 (—)	6 (—)	8 (—)	6 (—)	6 (—)	10 (—)	28 (—)	30 (—)
Tetracycline (TE)	Zone (mm)	14 (—)	17 (—)	19 (—)	13 (—)	15 (—)	15 (—)	14 (—)	14 (—)	10 (—)
Rifampin (RIF)	Zone (mm)	40 (—)	40 (—)	38 (—)	40 (—)	36 (—)	40 (—)	35 (—)	40 (—)	30 (—)
Amikacin (AK)	Zone (mm)	14 (—)	15 (—)	10 (—)	15 (—)	15 (—)	15 (—)	15 (—)	16 (—)	21 (—)
Chloramphenicol (C)	Zone (mm)	31 (—)	25 (—)	32 (—)	31 (—)	32 (—)	28 (—)	28 (—)	35 (—)	40 (—)

Abbreviations: MIC, minimum inhibitory concentration; S, susceptible; I, intermediate; R, resistant; —, interpretive category not assigned (no CLSI or EUCAST breakpoint for anaerobic bacteria/*Peptoniphilus* spp.). Susceptibility was determined by E-test (MIC, mg/L) for penicillin, ceftriaxone, meropenem, imipenem, vancomycin, and linezolid, and by disk diffusion (zone diameter, mm) for the remaining agents, on blood-supplemented Mueller–Hinton agar incubated anaerobically at 35–37 °C for 48 h. Interpretive categories were assigned using combined EUCAST v16.1 breakpoints for *Peptoniphilus* spp. as the primary reference. Where EUCAST provides no applicable breakpoint, CLSI M100 anaerobic breakpoints were used only when the breakpoint format matched the method performed. For cefepime, linezolid, erythromycin, levofloxacin, rifampin, and amikacin, neither EUCAST nor CLSI provides anaerobic breakpoints applicable to Peptoniphilus; therefore, only measured values are reported, and no interpretive category (—) was assigned for any isolate, including the reference strains.

## Data Availability

The 16S rRNA gene sequences generated in this study have been deposited in the GenBank database under accession numbers PX884293–PX884298 and PQ881956. The whole-genome sequencing raw reads have been deposited in the Sequence Read Archive (SRA) under accession numbers SRR40371697–SRR40371703. The associated BioProject accession number is PRJNA1519044, and the BioSample accession numbers are SAMN62712406–SAMN62712412. All other data generated or analyzed during this study are included in the published article and its Supplementary Materials.

## Supplementary Materials

The following supporting information can be downloaded at: https://www.mdpi.com/article/10.3390/pathogens15101073/s1, Figure S1: Gram staining (×1000 times) of all *Peptoniphilus genitalis*; Table S1: VITEK-2 ANC biochemical reactions of the seven clinical isolates and two reference strains; Table S2: 16S rRNA gene sequence comparison of the seven clinical isolates against reference sequences; Table S3: Genome assembly and annotation statistics of the seven *Peptoniphilus genitalis* isolates; Table S4: Average nucleotide identity (ANI) between the seven clinical isolates and reference genomes; Table S5: Antimicrobial susceptibilities of *Peptoniphilus genitalis* isolates in this study and of *Peptoniphilus* species in previous studies.
