# Supplementary material for: Whole-Genome Sequencing Re-Identifies Clinical “Peptoniphilus harei” Isolates as “Peptoniphilus genitalis”
Source: Pathogens. 2026 Oct 9;15(10):1073. doi: 10.3390/pathogens15101073 (PMC13708333; doi:10.3390/pathogens15101073)
Supplement: Supplementary file 1 [file pathogens-15-01073-s001.zip › pathogens-4560326-supplementary.pdf]

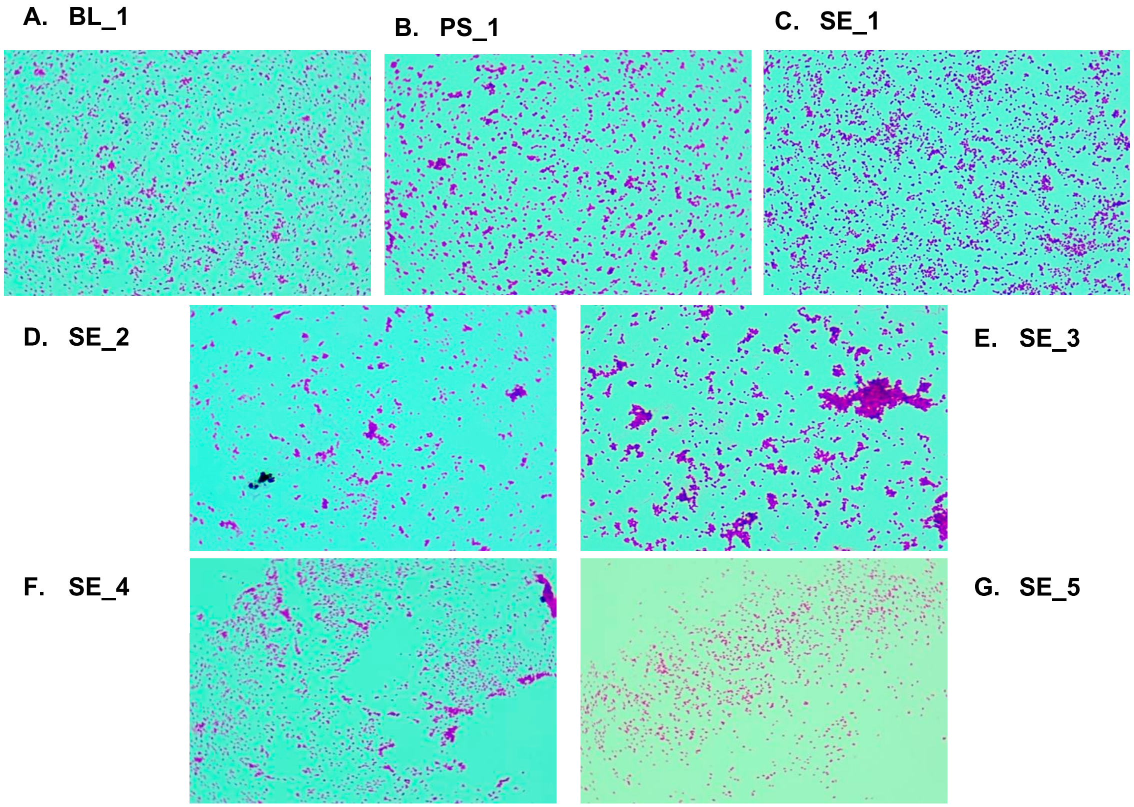

Figure S1. Gram staining (× 1000 times) of All *Peptoniphilus genitalis.*

Table S1. VITEK-2 ANC biochemical reactions of the seven clinical isolates and two reference strains.

Reaction	BL 1	PS 1	SE 1	SE 2	SE 3	SE 4	SE 5	DSM 101742	KCTC 5952	Reaction	BL 1	PS 1	SE 1	SE 2	SE 3	SE 4	SE 5	DSM 101742	KCTC 5952
D-Galactose	-	-	-	-	-	-	-	-	-	β-galactopyranoside indoxyl	-	-	-	-	-	-	-	-	-
Leucine arylamidase	-	+	-	-	+	+	-	-	-	α-arabinosidase	-	-	+	-	-	-	-	-	-
Ellman	+	-	-	-	+	+	-	+	-	5-Bromo-4-chloro-3-indoxyl-α-galactoside	-	-	-	-	-	-	-	-	-
Phenylalanine arylamidase	-	-	-	-	-	-	-	-	-	β-mannosidase	-	-	-	-	-	-	-	-	-
L-Proline arylamidase	-	-	-	-	-	-	-	-	-	Arginine	-	-	-	-	-	-	-	-	-
L-Pyrrolydonyl-arylamidase	-	-	-	-	-	-	-	-	-	Pyruvate	-	-	-	-	-	-	-	-	-
D-Cellobiose	-	-	-	-	-	-	-	-	-	Maltotriose	-	-	-	-	-	-	-	-	-
Tyrosine arylamidase	-	-	-	-	-	-	-	-	-	Esculin hydrolysis	-	-	-	-	-	-	-	-	-
Ala-Phe-Pro arylamidase	-	-	-	-	-	-	-	-	-	β-D-fucosidase	-	-	-	-	-	-	-	-	-
D-Glucose	-	-	-	-	-	-	-	-	-	5-Bromo-4-chloro-3-indoxyl-β-N-acetyl-glucosaminide	-	-	-	-	-	-	-	-	-
D-Mannose	-	-	-	-	-	-	-	-	-	5-Bromo-4-chloro-3-indoxyl-α-mannoside	-	-	-	-	-	-	-	-	-
D-Maltose	-	-	-	-	-	-	-	-	-	α-L-fucosidase	-	-	-	-	-	-	-	-	-
Saccharose/sucrose	-	-	-	-	-	-	-	-	-	Phosphatase	-	-	-	-	-	-	-	-	-
Arbutin	-	-	-	-	-	-	-	-	-	L-Arabinose	-	-	-	-	-	-	-	-	-
N-Acetyl-D-glucosamine	-	-	-	-	-	-	-	-	-	D-Ribose	-	-	-	-	-	-	-	-	-
5-Bromo-4-chloro-3-indoxyl-β-glucoside	-	-	-	-	-	-	-	-	-	Phenylphosphonate	-	-	-	-	-	-	-	-	-
Urease	-	-	-	-	-	-	-	-	-	α-L-arabinofuranosidase	-	-	-	-	-	-	-	-	-
5-Bromo-4-chloro-3-indoxyl-β-glucuronide	-	-	-	-	-	-	-	-	-	D-Xylose	-	-	-	-	-	-	-	-	-

+, positive; −, negative. a Results were generated by the VITEK-2 Compact system with the Anaerobic and Corynebacterium (ANC) identification card (bioMérieux). All seven clinical isolates and both reference strains were identified as P. asaccharolyticus by this card (confidence ≥98%). b DSM 101742 is the type strain of *P. vaginalis* (KHD2); KCTC 5952 is the type strain of *P. harei* (DSM 10020, SBH 432).

Table S2. 16S rRNA gene sequence comparison of the seven clinical isolates against reference sequences.

Isolate	Length (bp)	vs. *P. vaginalis* (NR_179440.1)	Gaps	vs. *P. harei* (NR_026358.1)	Gaps
BL 1	1,422	99.09% (1,409/1,422 nt)	3 (0.21%)	98.24% (1,397/1,422 nt)	4 (0.28%)
PS 1	1,429	98.60% (1,409/1,429 nt)	5 (0.35%)	98.04% (1,401/1,429 nt)	6 (0.42%)
SE 1	1,420	99.23% (1,409/1,420 nt)	3 (0.21%)	98.24% (1,395/1,420 nt)	4 (0.28%)
SE 2	1,420	99.01% (1,406/1,420 nt)	3 (0.21%)	98.31% (1,396/1,420 nt)	4 (0.28%)
SE 3	1,432	98.60% (1,412/1,432 nt)	4 (0.28%)	98.12% (1,406/1,433 nt)	7 (0.49%)
SE 4	1,421	98.87% (1,405/1,421 nt)	4 (0.28%)	98.03% (1,393/1,421 nt)	5 (0.35%)
SE 5	1,421	98.66% (1,402/1,421 nt)	4 (0.28%)	98.10% (1,394/1,421 nt)	5 (0.35%)

a Sequence identity, alignment length and gaps were determined by BLASTn search (NCBI 16S ribosomal RNA sequences database, default parameters, 19 August 2026) of each clinical isolate 16S rRNA gene sequence against the near-full-length reference sequences of P. vaginalis (type strain KhD-2, NR_179440.1) and P. harei (type strain DSM 10020, NR_026358.1). b Identities to P. vaginalis ranged from 98.60% to 99.23%, and identities to P. harei ranged from 98.03% to 98.31%. The P. vaginalis range straddled the 98.65% species-level threshold, whereas the P. harei range fell entirely below it. Neither range consistently exceeded the threshold, and no isolate could be confidently assigned to a species by 16S rRNA sequencing alone. c No 16S rRNA gene reference sequence of *P. genitalis* was present in the NCBI 16S database at the time of testing; therefore, a direct 16S comparison against *P. genitalis* was not possible, and the species assignment of the seven isolates relies on whole-genome sequencing and ANI.

Table S3. Genome assembly and annotation statistics of the seven *Peptoniphilus genitalis* isolates.

Isolate	Total reads (clean)	Q30 (%)	GC content (%)	Assembly size (bp)	No. of contigs	N50 (bp)	No. of CDS	No. of tRNA	No. of rRNA	Total genes
**BL 1**	6,731,146	96.44	34.97	1,959,528	31	241,497	1,797	37	5	1,797
**PS 1**	9,129,270	96.86	34.68	2,058,043	14	336,037	1,975	32	4	1,975
**SE 1**	8,395,832	97.49	34.72	1,948,787	121	79,273	1,761	32	2	1,761
**SE 2**	6,982,862	96.85	35.07	1,951,153	18	238,242	1,845	35	4	1,845
**SE 3**	9,126,996	96.54	34.72	1,968,727	13	274,260	1,826	27	4	1,826
**SE 4**	7,461,190	96.01	35.11	1,774,698	13	315,004	1,651	35	6	1,651
**SE 5**	6,776,286	96.46	35.18	1,835,649	17	285,065	1,718	36	4	1,718

Abbreviations: CDS, coding sequences; N50, the contig length at which 50% of the assembly is contained in contigs of equal or greater length; Q30, percentage of bases with a Phred quality score above 30. a Sequencing was performed on the Illumina platform (PE150); reads were quality-controlled with FastQC v0.11.2 and Trimmomatic v0.36; assemblies were generated with SPAdes v3.5.0 and gene prediction was performed with the NCBI Prokaryotic Genome Annotation Pipeline (PGAP v1.2.1). b Genome statistics were extracted from the sequencing company report (Shangong Biotech).

Table S4. Average nucleotide identity (ANI) between the seven clinical isolates and reference genomes.

Isolate	*P. genitalis* (GCF_023614525.1)	*P. vaginalis* (GCF_900176605.1)	*P. harei* (GCF_016726485.1)	Intra-cluster ANI range (%)	Species assignment
BL 1	97.05	92.15	89.32	96.71–98.20	***P. genitalis***
PS 1	96.44	91.84	89.02	96.71–98.20	***P. genitalis***
SE 1	96.70	92.08	89.32	96.71–98.20	***P. genitalis***
SE 2	96.71	92.29	88.92	96.71–98.20	***P. genitalis***
SE 3	96.64	91.97	89.32	96.71–98.20	***P. genitalis***
SE 4	96.93	91.98	89.03	96.71–98.20	***P. genitalis***
SE 5	96.92	91.99	88.86	96.71–98.20	***P. genitalis***

Abbreviations: ANI, average nucleotide identity; P., Peptoniphilus. a ANI values were computed using FastANI v1.33 with default settings (k-mer size 16, fragment length 3,000 bp, minimum fraction 0.2);; pairwise values between each clinical isolate and each reference genome are shown. The species boundary was set at 95% ANI. b Intra-cluster ANI range denotes the range of pairwise ANI values among the seven clinical isolates (96.71–98.20%), indicating that all isolates belong to a single species. c All seven isolates showed ANI values ≥95% against P. genitalis (96.44–97.05%) and <95% against P. vaginalis (91.84–92.29%) and P. harei (88.86–89.32%), confirming their assignment to P. genitalis.

Table S5. Antimicrobial susceptibilities of Peptoniphilus genitalis isolates in this study and of Peptoniphilus species in previous studies.

Antimicrobial agent a	Diop 2019 (P. vaginalis, 16S)	Wan 2021 (P. harei, 16S)	Cobo 2017 (P. harei, MALDI)	Cobo 2018 (P. harei, MALDI)	Cornman 2019 (P. harei)	Le Bihan 2019 (P. harei, MALDI)	Ueshima 2023 (P. harei, MALDI)	Abou Chacra 2024 (P. genitalis) [b]	This study[a] (7 isolates, P. genitalis)
Identification	P. vaginalis	P. harei	P. harei	P. harei	P. harei	P. harei	P. harei	P. genitalis	P. Genitalis
Identification method	16S rRNA	16S rRNA (MALDI failed)	MALDI-TOF	MALDI-TOF	N.A. (reference not located)	MALDI-TOF	MALDI-TOF	WGS (taxonogenomics)	WGS/ANI
Infection source	Vagina	Blood (aortic aneurysm)	Abdomen (peritoneum)	Lymphocele	Uterus/adnexa	Breast	Breast	Vagina	Blood/pus/secretion
AST method	E-test (MIC)	E-test (MIC)	MIC (method not stated)	MIC (method not stated)	Not stated	Culture/sensitivity (method not stated)	Not stated (qualitative)	E-test (MIC)	E-test (MIC) + disk diffusion
Breakpoint system (NEW)	EUCAST recommendations	CLSI (2021)	Not stated	Not stated	Not stated	Not stated	Not stated	EUCAST recommendations	EUCAST v16.1; CLSI M11 (MIC only)

Result unit	MIC (μg/mL)	MIC (μg/mL)	MIC (mg/L)	MIC (mg/L)	Qualitative	Qualitative	Qualitative	MIC (μg/mL)	MIC (mg/L) and zone (mm)
Penicillin	0.094 (S) [c]	0.032 (S)	0.19 (S)	0.064 (S)	S	/	S	<0.016 (S) [d]	0.006–0.25 (S)
Amoxicillin	0.032 (S)	/	/	/	/	/	/	0.023 (S) [d]	/
Amoxicillin-clavulanate	/	/	0.064 (S)	0.064 (S)	/	/	/	/	38–42 mm (S)
Piperacillin-tazobactam	/	/	0.016 (S)	0.016 (S)	/	/	/	/	40–48 mm (S)
Ceftriaxone	0.064 (S)	0.125 (S)	/	/	/	/	S	0.47 (S)	0.094–0.38 (S)
Cefepime	/	/	/	/	/	/	/	/	11–34 mm (—)
Meropenem	/	0.008 (S)	0.012 (S)	0.006 (S)	/	/	/	/	0.006–0.04 (S)
Imipenem	0.004 (S)	/	0.032 (S)	0.008 (S)	/	/	/	0.004 (S)	0.008–0.024 (S)
Vancomycin	0.094 (S)	/	0.125 (S)	0.064 (S)	/	/	/	0.064 (S)	0.125–0.19 (S)
Linezolid	/	/	0.75 (S)	0.25 (S)	/	/	/	0.75 (—)	0.125–0.5 (—)
Erythromycin	1 (R*)	/	/	/	/	/	/	azithromycin 0.5 (—) [e]	6–21 mm (—)
Clindamycin	/	0.064 (S)	0.5 (S)	0.094 (S)	R (unverified)	/	/	0.5 (S)	6–26 mm; 6/7 R
Levofloxacin	/	/	/	/	/	/	/	/	6–21 mm (—)
Moxifloxacin	/	/	0.75 (S)	0.19 (S)	/	/	/	/	6–32 mm (—)
Tetracycline	/	/	/	/	/	/	/	doxycycline 1.5 (—) [e]	13–19 mm (—)
Rifampin	0.002 (S)	/	/	/	/	/	/	0.002 (—)	35–40 mm (—)
Amikacin	>256 (R[c])	/	/	/	/	/	/	no activity (—)	10–15 mm (—)
Chloramphenicol	/	1.5 (S)	/	/	/	/	/	/	25–32 mm (—)
Metronidazole	0.125 (S)	0.064 (S)	6 (S)	1.5 (S)	S (unverified)	S	/	0.5 (S)	not tested

Abbreviations: MIC, minimum inhibitory concentration; S, susceptible; I, intermediate; R, resistant; N.A., not available; /, not tested.; —, interpretive category not assigned (no CLSI or EUCAST anaerobic breakpoint for Peptoniphilus spp.).a For "This study", MICs were measured by E-test and zone diameters (mm) by disk diffusion. Interpretive categories followed EUCAST v16.1 (Peptoniphilus spp.) where available; ceftriaxone (MIC) used the CLSI M11 MIC breakpoint; because CLSI provides MIC, not disk-diffusion, breakpoints for anaerobes, agents tested by disk diffusion only without an EUCAST zone breakpoint (moxifloxacin, tetracycline, chloramphenicol) and agents lacking an applicable anaerobic breakpoint (cefepime, linezolid, erythromycin, levofloxacin, rifampin, amikacin) are reported without interpretation (—). Previous studies reported MIC (E-test or broth) or qualitative susceptibility rather than disk-diffusion zone diameters, so their values are not directly method-comparable with the zone-based data herein. R* indicates an S/R label assigned by the original authors although no EUCAST/CLSI anaerobic breakpoint is available for that agent in Peptoniphilus. b Wang et al. 2022 described a blood-culture isolate (SAHP1) closely related to P. vaginalis (provisionally P. septimus) from a cervical cancer patient; that report did not provide isolate-level susceptibility data, so it is not listed as a numeric column here. c Diop et al. 2019 tested benzylpenicillin (not ampicillin/penicillin) and amoxicillin (not amoxicillin-clavulanate); they also tested ertapenem (0.002, S) and ofloxacin (>256, reported R; no EUCAST anaerobic breakpoint) d Abou Chacra et al. 2024 tested penicillin G and amoxicillin (MIC, μg/mL). e In Abou Chacra et al. 2024, azithromycin and doxycycline were tested instead of erythromycin and tetracycline, respectively.
